# Supplementary material for: Are Basic Substances a Key to Sustainable Pest and Disease Management in Agriculture? An Open Field Perspective
Source: Plants (Basel). 2023 Sep 1;12(17):3152. doi: 10.3390/plants12173152 (PMC10490370; doi:10.3390/plants12173152)
Supplement: Supplementary file 1 [file plants-12-03152-s001.zip › plants-2570959-supplementary.pdf]

Table S1: Comprehensive resume of approved basic substances according to the European pesticide database (accessed 28 March 2023). Type of product that employed its function and situation of use are also reported.

Approved basic substance	Chemical family, group or nature	Type of product	Function of plant protection	Crop or situation	FGI[a]	Pest or disease controlled	Way of application
Allium cepa L. bulb extract	Water extract from plant tissues	Dispersible concentrate (DC) obtained from decoction of crude onion	Fungicide	Potato (*Solanum tuberosum*)	F	Early blight	Spray application
				Tomato (*Solanum lycopersicum*)	FG	Late Blight	
				Cucumber (*Cucumis Sativus*)	FG	Gray Mold	
Beer	Food product	Undiluted	Molluscicide	All edible and non-edible crops	F	Pest slugs and snails	Used in covered slug traps
Calcium hydroxide	Inorganic salt (base)	Liquid suspension (acqueous)	Fungicide	Pome fruit	F	*Neonectria galligena*	Splinker
				Pome fruit and stone fruit	F	*N. galligena* and other diseases	Spray application
				Pome fruit and stone fruit	F	N. galligena and other diseases	Direct application on wounds and cancers
Chitosan	Polycationic polysaccharide	Soluble powder (SP) to be diluted in water	Plant elicitor	Olive trees (*Olea europaea*)	F	Plant resistance against pathogenic fungi and bacteria	Spray application
				Grapevine (*Vitis vinifera*)	F		
				Grass, grasslands and ornamental grasses	FG		
				Grass (Sport fields, Golf courses)	FG		
				Ornamental plants	FGI		
				Post-harvest fruit treatment	FG		Immersion/dipping
				Fruits berries and small fruit, vegetables, cereals, spices,	FG		Low volume spray or dipping

				crops for animal feed			
				Cereals, potatoes, sugar beet	FG		
Chitosan hydrochloride	Linear polysaccharide composed of randomly distributed 1-4 linked D glucosamine and N-acetyl-D-glucosamine produced by de-acetylation of chitin	Soluble powder (SP) to be diluted in water	Plant elicitor	Fruits berries and small fruit, vegetables, cereals, spices, crops for animal feed	FG	It stimulates plant resistance against pathogenic fungi and bacteria	Low volume spray or dipping
				Cereals, potatoes, sugar beet	FG		
				Ornamental bulbous	FGI		
				Beet crops	F		
Clayed charcoal	Mixture of charcoal and bentonite	Granule (GR)	Protectant	Grapevine (V. vinifera)	F	ESCA disease causing complex	Soil burying
Cow Milk	Food product	Soluble concentrate (SL)	Fungicide and virucide	Grapevine (V. vinifera)	F	Powdery mildews	Foliar application
				Tomato (S. lycopersicum)	G		
				Gerbera (*Gerbera jamesonii*)	G		
				Cucumber (*C. sativus*) and Zucchini squash (*Cucurbita pepo*)	G		
				Soybean (*Glycine max*)	F		
				Glove fingertips and mechanical cutting tools		Viruses	Dipping
Diammonium phosphate	Inorganic salt	Soluble powder (SP) to be diluted in water	Insect attractant	Orchards, including cherry tree and *Prunus spp.*	F	Mediterranean fruit fly, cherry fly	Placed in physical traps
				Olive trees (*O. europaea*)	F	Olive fly	

				Citrus spp.	F	Mediterranean fruit fly	
				Other crops	F	Mediterranean fruit fly	
Equisetum arvense L.	Water extract from plant tissues	Dispersible concentrate (DC) obtained from decoction of *Equisetum arvense* L. dry plant (2 g/L in the final preparation)	Fungicide	Fruit trees (apple tree and peach tree)	F	Foliar fungi like scab and peach leaf curl	Foliar application
				Grapevine (*V. vinifera*)	F	Downy and powdery mildews	
				Cucumber (*C. sativus*)	G	Powdery mildews and root rot fungi	
				Tomato (S. lycopersicum)	F	Early blight and *Septoria* blight	
		Dispersible concentrate (DC) obtained from decoction of *Equisetum arvense* L. dry plant (2.25 g/L in the final preparation)		Strawberry (*Fragaria* x *ananassa*) and Raspberry (*Rubus idaeus*)	FG	Grey mold, powdery mildew, red core and other fungi	
				Potato (*S. tuberosum*)	FG	Late blight, early blight and powdery mildew	
		Preparation to be used as mulch incorporation		Cucumber (*C. sativus*)	GF	Powdery mildes, root rot fungi and seedling blight	Dry, Included in mulch
				Tomato (S. lycopersicum)	F	Early blight and *Septoria* blight	
				Ornamental plants (including Prunus spp.)	FG	Rose black spot, rose rust and leaf curl	
Fructose	Monosaccharide	Soluble powder (SP) to be diluted in water	Plant elicitor	Apple trees (*Malus spp*.)	F	Fruits borer like codling moth	Foliar application (early in the morning)
				Maize (*Zea mays*)	F	Symphylans	
				Grapevine (*V. vinifera*)	F	Vine leafhopper	

				Grapevine (*V. vinifera*)	F	Downy mildew	
Hydrogen peroxide	Peroxide	Liquid for disinfection of agricultural mechanical cutting tools	Fungicide and bactericide	Vegetables, Solanaceae spp.	G	Soil bacteria, grey mold	Apply before cutting
		Liquid for seed treatment (LS)		Lettuce (*Lactuca sativa*) and horticulture flowers	FG	Bacterial leaf spot and pathogenic fungi	Seed treatment before sowing
L-cysteine	Proteinogenic amminoacid	To be used in a mixture with matrix (wheat flour, food grade) at a concentration of maximum 8%	Insecticide	All crops and forestry (in tropical areas)	F	Leaf cutting ants	Hand-held spreader
Lecithins	Food additive	Emulsiafiable concentrate (EC)	Fungicide	Fruit trees (apple tree and peach tree)	FG	Powdery mildews	Spray application
				Gooseberry (*Ribes uva-crispa*)	FG		
				Market vegetables (e.g. *C. sativus*)	FG		
				Lettuce (*Lactuca sativa*)	FG		
				Mash (*Valerianella locusta*)	FG		
				Tomato (S. lycopersicum)	FG	Late blight	
				Endive (*Cichorium endivia*)	FG	*Alternaria cichorii*	
				Ornamentals (especially roses)	FG	Powdery mildews and other fungal diseases	
				Grapevine (*V. vinifera*)	F	Downy and powdery mildews	

				Strawberry (*F. x ananassa*) and Raspberry (*R. idaeus*)	FG	Powdery mildews and red core	
				Potato (*S. tuberosum*)	FG	Late blight	
				Carrot (*Daucus carota* subsp. *sativus*)	FG	Powdery mildew	
Mustard seeds powder	Dry extract from plant tissues	Water dispersible powder for slurry seed treatment (WS)	Fungicide	Weath seeds	F	Common blunt (*Tilletia* spp.)	Seed application before sowing
Onion oil	Food product	Oil dispersion (OD)	Repellent and scent masking	Umbelliferous crops (e.g. carrots, celeriac, parsnip, parsley root)	F	Carrot root fly	Evaporated from dispenser
Salix spp. cortex	Water extract from plant tissues	Dispersible concentrate (DC) from bark of Salix	Fungicide	Fruit trees and peach trees	F	Foliar fungi (e.g. *Taphrina deformans*)	Seed application before sowing
				Apple trees (*Malus* spp.)	F	Scab disease and powdery mildew	
				Grapevine (*V. vinifera*)	F	Downy and powdery mildews	
Sodium chloride	Inorganic salt	Water soluble powder (SP)	Fungicide	Grapevine (*V. vinifera*)	F	Powdery mildew	Foliar application
		Granule (GR)		Mushrooms (e.g. *Agaricus bisporus*)	G	Fungal diseases	
		Water soluble powder (SP)	Insecticide	Grapevine (*V. vinifera*)	F	European grapevine moth	
			Herbicide	Salt swamps and salt marshes	F	*Baccharis halimfolia*	
Sodium hydrogen carbonate	Inorganic salt	Water soluble powder (SP)	Fungicide	Vegetables, soft fruits and ornamentals	FG	Mildews (e.g. Sphaerotheca and Oidium spp.)	Foliar application

				Grapevine (*V. vinifera*)	F	Powdery mildew	
				Apple trees (*Malus spp.*)	F	Apple scab	
				Fruits (e.g. oranges, cherries, apples, papaya)	FI	Storage diseases (e.g. *Penicillium spp.*)	Dipping or surface treatment
		Granule (GR)	Herbicide	Potted plants	G	Liverwort, bryophyte and green thallus of liverwort)	Direct application
Sucrose	Disaccharide	Water soluble powder (SP)	Plant elicitor	Apple trees (*Malus spp.*)	F	Fruits borer like Codling moth	Foliar application (early in the morning)
				Maize (*Zea mays*)	F	Corn borer	
				Grapevine (*V. vinifera*)	F	Vine leafhopper and downy mildew	
Sunflower oil	Food product	Oil dispersion (OD)	Fungicide	Tomato (S. lycopersicum)	F	Powdery mildew	Foliar application
Talc E553B	Silicate mineral	Wettable powder (WP)	Fungifuge	Grapevine (*V. vinifera*)	F	Powdery mildew	Spray application
				Apple (*Malus spp.*) and Pear (*Pyrus spp.*) tree	F	Apple scab	
			Insectifuge	Fruit trees (*Malus*, *Pyrus spp.*) and Olive tree (*O. europea*)	F	Insects and mites	
Urtica spp.	Water extract from plant tissues	Dispersible concentrate (DC)	Insecticide	Fruit trees (*Malus*, *Pyrus spp.*), redcurrant (*R. rubrum*) and walnut tree (*Juglans spp.*)	F	Aphids	Foliar or direct application
				Bean (*Phaseolus vulgaris*)	F		
				Potato (*S. tuberosum*)	F		

				Leaf vegetables (e.g. *Lactuca spp.*) and cabbages (*Brassica spp.*)	F		
				Elder tree (*Sambucus racemosa*)	F		
				Rose (*Rosa spp.*)	F		
				Spiraea spp.	F		
				Brassicaceae (*Brassica spp.*)	F	Fleabeetle and diamond back moth	Foliar application
				Apple tree (*Malus domestica*) and Peer tree (*Pyrus communis*)	F	Codling moth	
			Acaricide	Bean (*P. vulgaris*)	F	Two-spotted spider mite	
				Grapevine (*V. vinifera*)	F	Two-spotted spider mite	
			Fungicide	Brassicaceae (mustard family)	F	Alternaria spp.	Foliar and fruit application
				Cucumber (*C. sativus*)	F	Alternaria spp. and powdery mildew	
				Fruit trees (e.g. Apple, Plum, Peach and Sweet cherry tree)	F	Leaf spot, brown rot, grey mold and black bread mold	
				Grapevine (*V. vinifera*)	F	Downy mildew	
				Potato (*S. tuberosum*)	F	Late blight	
Vinegar	Food product	Liquid for seed treatment (LS)	Fungicide	Wheat (*Triticum spp.*) seeds	F	Common blunt (e.g. Tilletia spp.)	Seed treatment before sowing
				Barley (*Hordeum vulgare*) seeds	F	Barley leaf stripe	

				Market vegetables	F	Alternaria spp.	
			Bactericide	Market vegetables	FG	Bacterial borne diseases	
		Liquid for disinfection of mechanical cutting tools (LS)	Bactericide	White and red chestnut and Acer spp.	F	Pseudomonas syringae pv aesculi	Tools application before sawing or cutting
				Ornamental plants	F	Fire blight	
					F	Bacterial blight/canker	
				Plane (*Platanus spp.*), *Prunus spp.*, Chestnut (*Aesculus spp.*), *Sophora spp.*, Linden (*Tilia spp.*)	F	Rot fungi	
			Fungicide	Elm tree (*Ulmus spp.*)	F	Vascular fungi (*Opiostoma spp.*)	
				Maple tree (*Acer spp.*)	F	Wilt disease	
				Ailanthus altissima	F	*Verticillium spp.*	
				Maple (*Acer spp.*), chestnut (*Aesculus spp.*) and beech (*Fagus spp.*)	F	Sooty-Bark disease	
Whey	Food product	In water solution	Fungicide and virucide	Cucumber (*C. sativus*) and Zucchini squash (*C. pepo*)	G	Powdery mildews	Spray application
				Grapevine (*V. vinifera*)	F	Powdery mildew	
				Tomato (S. lycopersicum)	FG	Tomato yellow leaf curl virus	
				Glove fingertips and mechanical cutting tools		Viruses	Dipping

[a] Outdoor or field use (F), greenhouse application (G) or indoor application (I).
